# Experience with Dopamine Agonists in the Treatment of Prolactinomas

**DOI:** 10.15388/Amed.2022.29.2.15

**Published:** 2022-06-29

**Authors:** Nadiya Ye. Barabash, Tetiana M. Tykhonova

**Affiliations:** V. N. Karazin Kharkiv National University, Kharkiv, Ukraine; V. N. Karazin Kharkiv National University, Kharkiv, Ukraine

**Keywords:** hyperprolactinemia, dopamine agonists, cabergoline, dopamine resistance

## Abstract

The article is devoted to the conservative treatment of hyperprolactinemia of tumor origin. Cabergoline is considered as an effective treatment not only for microadenomas but also for large pituitary tumors which is illustrated by the clinical case of the patient P. The most important effects of cabergoline demonstrated in this clinical case are reduction in the size of the adenoma from macro to micro, reverse development of chiasmal syndrome with restoration of visual functions, achievement and maintenance of the target level of prolactin. Besides the article focused on the possible development of primary and secondary resistance to dopamine analogues. One of such difficult clinical scenarios is illustrated by the clinical case of patient M., who was treated with different dopamine analogues with the development of secondary dopamine resistance. Along with described in literature possible mechanisms for its development in our patient we also suggest the role of prolonged inadequate therapy with dopamine agonists, when the dose of the drug was not properly adjusted, despite not reaching the target prolactin level. The literature considers several ways of optimization of treatment in resistant patients but despite of this fact it remains one of unresolved problems in the management of patients with hyperprolactinemia.

## Santrauka.

Šiame straipsnyje aprašomas konservatyvus vėžinės kilmės hiperprolaktinemijos gydymas. Kabergolinas (*Cabergoline*) yra veiksminga priemonė ne tik mikroadenomoms, bet ir dideliems hipofizės navikams gydyti. Tai iliustruojama paciento P klinikiniu atveju. Svarbiausias kabergolino poveikis šiuo klinikiniu atveju yra tas, kad adenoma sumažėjo nuo makro- iki mikrodydžio atgalinės krypties chiasma sindromo atveju. Buvo atkurta regėjimo funkcija bei pasiektas ir palaikomas tikslinis prolaktino lygis. Be to, straipsnyje sutelkiamas dėmesys į pirminę ir antrinę rezistenciją dopamino analogams. Vienas iš tokių sudėtingų klinikinių atvejų iliustruojamas klinikiniu paciento M atveju. Šis pacientas buvo gydomas įvairiais dopamino analogais. Išsivystė antrinė rezistencija dopaminui. Kartu su literatūroje aprašytais galimais mechanizmais, taikytais mūsų pacientui, taip pat siūlome aptarti ištęstos neadekvačios dopamino agonistų terapijos, kai vaisto dozė nebuvo tinkamai nustatyta, nepaisant to, kad nebuvo pasiektas tikslinis prolaktino lygis, poveikį. Literatūroje svarstoma keletas būdų efektyvinti dopaminui atsparių pacientų gydymą, tačiau pacientų, kuriems yra hiperprolaktinemija, tinkama gydymo strategija tebėra viena iš neišspręstų problemų.

## Introduction

According to statistics hyperprolactinemia occurs in less than 1% of the general population [1] but its incidence is steadily increasing and is one of the main causes of endocrine infertility [2]. One of the possible genesis of hyperprolactinemic syndrome is tumor. Among all pituitary adenomas, prolactinoma is the most common type of tumor and occurs in about 40% of cases. If a pituitary tumor is present, it is a microadenoma (< 10 mm) in approximately 90% of cases [3].

According to the Endocrine Society Clinical Practice Guideline for the Diagnosis and Treatment of Hyperprolactinemia 2011 [4] which wasn’t changed during the last 11 years the method of choice in the treatment of hyperprolactinemia in symptomatic patients with microadenomas or macroadenomas is conservative drug therapy with dopamine agonists. These drugs aim to decrease prolactin (PRL) levels, reduce tumor size and normalize reproductive function. Such therapy is indicated not only for small tumors but the effective use of cabergoline is described also in the treatment of giant prolactinoma [5].

One of the unresolved problems in the management of patients with hyperprolactinemia includes primary or, less frequently, secondary resistance to dopamine analogues, when standard doses of drugs fail to normalize the level of PRL and reduce tumor size by at least 50%. The inability to restore fertility with standard doses of agonists may also be a reflection of resistance to treatment. Every such case is a challenge for the doctor, because it is a difficult clinical scenario [6].

We report here two cases to show effectiveness of the treatment with cabergoline agonists in different clinical situations.

## Case 1

Patient P., 58 years old, was hospitalized to the endocrinological clinic due to the detection of a high level of PRL along with other complaints described below.

It is known that patient was a participant in hostilities, who received a concussion, shrapnel wounds to the skull and typhoid fever in his youth.

For 2 months before going to the doctor, the patient developed general weakness, loss of appetite, pain in the right side of the abdomen, for 2 weeks he lost about 10 kg in weight for no apparent reason. Gastroenterologist diagnosed erosive gastroduodenitis, acute stage, associated with Helicobacter pylori; billiary type Oddy’s sphinсter dysfunction, cholelithiasis, chronic calculous cholecystitis, chronic colitis. Prescribed therapy did not lead to an improvement in well-being, general weakness progressed. Abdominal computed tomography revealed lymphadenopathy of the iliac lymph nodes on the left, a change in the diameter of the rectum lumen. Therefore the patient was referred to an oncologist with a suspicion of a malignant tumor of the gastrointestinal tract. Clinical, laboratory and instrumental examination did not reveal any evidence of oncological pathology. However, during the examination period, a short-term loss of consciousness was noted once, after which the patient’s hearing deteriorated, a “veil” appeared in front of his left eye. Taking into account these symptoms, a magnetic resonance image (MRI) of the brain was performed. A tumor formation of the sellar-chiasmal region 18.3x12.0x15.4 mm size with a clear, even contour, located intra-suprasellarly, with cranial displacement of the chiasm was revealed. Further examination found hyperprolactinemia (112.17 ng/ml, normal values are 5–25 ng/ml), a decrease in blood cortisol level to 21.02 nmol/l (normal values are 150–660 nmol/l), hypothyroidism (free thyroxine – 0.5 ng/dl, normal values are 0.7–1.48 ng/dl, thyroid stimulating hormone – 0.04 mIU/ml, normal values are 0.4–4.0 mIU/ml), hypogonadotropic hypogonadism (luteinizing hormone (LH) – 1.0 mIU/ml, normal values are 1.7–11.2 mIU/ml; follicle stimulating hormone (FSH) – 4.3 mIU/ml, normal values are 2.1–18.6 mIU/ml; testosterone – 0.08 ng/ml, normal values are 2.62–8.7 ng/ml).

Taking into account the presence of a large tumor of the pituitary gland producing PRL, the presence of secondary insufficiency of the adrenal, thyroid glands and gonads, the following diagnosis was made: “Pituitary adenoma (macroprolactinoma). Hypopituitarism: secondary adrenal insufficiency, secondary hypothyroidism, hypogonadotropic hypogonadism”. The suppressive therapy with cabergoline at a dose of 0.5 mg/week, hormonal replacement therapy with prednisolone 10 mg/day, levothyroxine 50 mcg/day was prescribed. In addition, during the examination, an acute circulatory disorder in the optic nerve of both eyes was revealed with temporal visual field loss and involvement of the retinal fovea on the left and an upper temporal visual field loss on the right, as a manifestation of chiasmal syndrome. A course of therapy was carried out. As a result of the prescribed hormonal replacement therapy with levothyroxine and prednisolone, as well as suppressive treatment with cabergoline, the patient’s state of health improved significantly. After 6 months from the start of cabergoline therapy, positive dynamics was noted. According to MRI data the size of the pituitary gland decreased to 10.0x10.3x15.4 mm, its upper contour stick to the chiasm without signs of compression; the MR signal from the structure of the pituitary gland is close to normal.

## Case 2

Patient M., aged 43, has been suffering from hyperprolactinemia for a long time, and therefore was under observation. At the age of 20 she became physiologically pregnant and gave birth to a healthy boy, breastfeeding continued for 9 months. At the age of 24, due to the appearance of discharge from the mammary glands (3rd degree galactorrhea), an examination was carried out. As a result the patient was diagnosed with hyperprolactinemia, which exceeded the threshold of normal values by 1.3 times (29.4 ng/ml). For 8 months, she received therapy with the dopamine receptor agonist bromocriptine at an average therapeutic dose (2.5 mg 2 times a day) with a positive effect (normalization of the PRL level and the disappearance of galactorrhea). On the recommendation of the doctor, the patient stopped taking the drug. Over the next 4 years, the patient was worried about irregular menstruation, cycle lengthening up to 54–56 days, infertility, but she did not apply to a medical institution. At the age of 28 the excess of the PRL level was 2.7 times of norm (69.2 ng/ml). Since that time, the patient has been prescribed cabergoline at a dose of 0.5 mg/week. When analyzing the PRL over the next year, its constantly elevated level (up to 75.6 ng/ml) attracted attention, while the dose of cabergoline was increased not more than 1 mg/week. Only when hyperprolactinemia reaches 97.2 ng/ml (excess by 3.9 times), the dose of cabergoline was increased simultaneously to 1.75 mg/week (3.5 tablets). In this case, the level of PRL decreased, but did not fall below 23.9 ng/ml, despite the high dose of the drug. After a year of such therapy, the patient was recommended to gradually reduce the dose of cabergoline, against which the PRL value increased again and reached 113.4 ng/ml. Periods during this time were regular, but scanty, pregnancy did not occur. After 1.5 years without treatment, cabergoline therapy was resumed at a maximum dose of 2 mg/week. Six months later, when PRL decreased to 24 ng/ml, the patient noted recurring menorrhagia and severe mastodynia, from which she woke up at night. Cabergoline, due to insufficient efficacy, was replaced by quinagolide at an increasing dose up to the maximum. During this therapy the decrease in PRL level was not lower than 20.0 ng/ml. Subsequently, the patient canceled all drugs, while PRL ranged from 75.6 to 151.1 ng/ml.

For 15 years, only radiography of sella turcica was used as imaging research method, and it did not reveal pathological changes. Only at the age of 39 (15 years after the first detection of hyperprolactinemia) MRI of the brain was performed for the first time. It registered signs of a pituitary microadenoma of size 4x7. The administration of cabergoline 1.5 mg/week was resumed, which reduced the level of PRL till 53.46 ng/ml.

**Figure 1.**
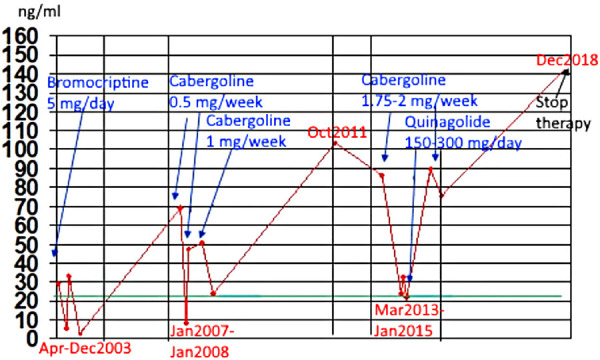
PRL dynamics in patient M.

The dynamics of PRL over the period from 2003 to 2018 is shown in Fig. 1.

## Discussion

The first clinical case is an illustration of the high effectiveness of cabergoline in large prolactinoma with symptoms of compression. One of them was chiasmal syndrom which lead to the visual disturbances. Also the hypopituitarism (secondary hypothyroidism, secondary adrenal insufficiency, and hypogonadism) that developed in the patient seems to be associated with mechanical compression by the tumor tissue of the adjacent pituitary structures which produce the corresponding tropic hormones. At the same time, the nature of hypogonadism is likely to be dual – associated with low levels of LH and FSH due to the above mentioned mechanism, and with hyperprolactinemia itself.

The most important effects of cabergoline demonstrated in this clinical case are:

1) reduction in the size of the adenoma from macro to micro;
2) reverse development of chiasmal syndrome with restoration of visual functions;
3) achievement and maintenance of the target level of PRL.

The detection of hyperprolactinemia in this patient at the stage of macroadenoma may be related to gender. In women, hyperprolactinemia most often manifests itself with a number of clear signs: disruption of the menstrual cycle, galactorrhea, infertility, etc. These symptoms make a woman see a doctor, as a rule, at the stage of microadenoma or without revealing an organic pathology of the pituitary gland according to MRI. In men, due to the absence of a clear clinical picture in the early stages of the disease, hyperprolactinemia remains undiagnosed for a long time, until the development of symptoms associated with hypogonadism (primarily erectile dysfunction) or chiasmatic syndrome. This leads to the fact that long-term hyperprolactinemia causes progressive hypertrophy of pituitary cells with the eventual formation of a macroadenoma, that is, a tumor of more than 1 cm in diameter.

In contrast to the first clinical case, the second one shows no effect of therapy despite the small size of the pituitary tumor.

When analyzing the dynamics of PRL during 16 years (fig. 1), attention is drawn to the so-called “escape” of the drug therapy effect, manifested by the phenomenon of secondary resistance to the treatment by dopamine agonists. Its essence in this patient was manifested in the fact that at the beginning of the treatment with this group of drugs, there was a decrease in the level of PRL, followed by a persistent increase of this hormone, despite the use of sufficiently high doses. At the same time, a similar phenomenon was observed sequentially with the prescription of bromocriptine, then cabergoline, then quinagolide.

For the entire period of treatment, the patient received treatment with cabergoline for the longest time. The highest dose used in this patient was 2 mg/week. The same dose was considered to be maximally tolerated, as the patient noted that she felt better without therapy. The use of dopamine agonists was accompanied by soreness of the mammary glands, the presence of period pains, and frequent headaches. Thus, the impossibility of achieving normal PRL level on the background of the use of the maximum tolerated doses of dopamine agonists became the basis for establishing the presence of secondary dopamine resistance.

Possible mechanisms that play role in resistance to dopamine agonists include decreased expression of dopamine receptors in tumor cells, changes in other receptors that modulate dopamine receptors (e.g., nerve growth factor receptor), changes in downstream cascades (e.g., in subunit of the G-protein), an increase in angiogenesis markers, and an increase in fibrosis due to disturbances in the transforming growth factor β1 pathway [4]. At the same time, prolonged inadequate therapy with dopamine agonists, when the dose of the drug was not properly adjusted, despite not reaching the target PRL level in the lower third of the reference range, can also be considered as one of the factors.

In this context, it should be emphasized that there are several approaches to prescribing an initial dose of cabergoline to a patient with hyperprolactinemia, in particular prolactinoma. The first approach involves the prescription of a starting dose of 0.25–0.5 mg per week, followed by a gradual increase in the dose of the drug every 4 weeks under the control of PRL levels in the blood. According to the instructions to the drug, the average therapeutic dose is 0.5–2 mg per week. The lack of effect when using 3 mg of cabergoline per week, as most authors say, indicates the inexpediency of further increasing the dose of the drug. However, other researchers believe that in some patients with micro- and macroprolactinoma the dose of cabergoline can reach 9 mg per week [7-8]. The preservation of the therapeutic effect in combination with the tolerability of the drug by patient and the absence of side effects should be considered as the main factors in deciding whether to increase the dose of the drug. If, while increasing the dose, there is a further decrease in the level of PRL, a tendency to reduce the size of prolactinoma, and there are no pronounced side effects, this tactic should be considered appropriate [9].

In some literature sources another approach to determining the initial dose of cabergoline is described. It involves the prescription of a high suppressive dose of the drug, taking into account the basal level of serum PRL [10]. The author of this mode of therapy believes that the appointment of large doses of the drug in women with hyperprolactinemiс syndrome prevents and further does not develop resistance to dopamine agonists. At the same time, it must be borne in mind that this approach is not supported by other evidence-based reviews or guidelines for the management of patients with hyperprolactinemia.

Summarizing the above, dopamine agonists are highly effective in most cases of treatment of hyperprolactinemia, in particular prolactinoma. At the same time, some patients may respond poorly to such treatment – they may be primary or secondary resistant to drug therapy. There is still no universal consensus on the determination of resistance to dopamine agonists, but the most universal is its definition as failure to achieve normoprolactinemia at the maximum tolerated dose for at least 3–6 months without reducing tumor diameter by at least 30% in macroprolactinoma [11]. There is also no clear answer as to which dose of dopamine agonist should be considered ineffective. Most often, and given the accepted dose equivalences between different drugs of this group, these doses are usually defined as ≥ 15 mg bromocriptine per day, ≥ 2.0 mg cabergoline per week and ≥ 225 mcg quinagolide per day. Resistant prolactinomas are most common with bromocriptine (approximately 20–30%) [12-13] and much less often for cabergoline, which remains the drug of choice in the treatment of hyperprolactinemia [14].

Despite of the fact that the main prognostic adverse factors for dopamine resistance are considered to be tumor invasiveness and male gender, in the presented clinical case there is a woman with microadenoma. Conversely, in the male patient with macroadenoma cabergoline therapy has been shown to be highly effective.

The literature considers several ways of optimization of the treatment in resistant patients [11]:

1) switching to cabergoline if the patient is treated with bromocriptine or quinagolide;
2) increasing the dose of cabergoline to the maximum tolerated;
3) increasing the dose of cabergoline to 3.5 mg per week and waiting;
4) surgery;
5) radiation therapy;
6) use of synthetic cytostatic antitumor drug temozolomide in case of very aggressive tumors.

## Conclusion

1. At present, in the management of patients with hyperprolactinemia physicians use the recommendations of 2011, which determine therapeutic approaches. At the same time, despite the existence of the algorithms, in each clinical case, a patient-centered approach to treatment is needed. This is due to the fact that along with the “classic” course of the pathology there are situations that are challenging for the doctor and require careful analysis and individual approach.
2. According to the recommendations, the method of choice in the treatment of hyperprolactinemia, in particular prolactinoma, is conservative therapy with dopamine agonists, primarily cabergoline due to its better efficacy, fewer side effects and more convenient mode of administration, unlike other drugs of this group. Cabergoline is also an effective method of reducing the size of prolactinomas, including large ones, as demonstrated by the clinical case.
3. One of the most difficult scenarios in the treatment with dopamine agonists is the presence of primary or the development of secondary dopamine resistance. Thus the decision of the further therapy depends on each specific clinical situation and require doctor’s considerable experience and proficiency.
